# Biogenic polymer-based patches for congenital cardiac surgery: a feasibility study

**DOI:** 10.3389/fcvm.2023.1164285

**Published:** 2023-06-22

**Authors:** Emma Richert, Andrea Nienhaus, Silje Ekroll Jahren, Amiq Gazdhar, Maximilian Grab, Jürgen Hörer, Thierry Carrel, Dominik Obrist, Paul Philipp Heinisch

**Affiliations:** ^1^ Department of Congenital and Paediatric Heart Surgery, German Heart Centre Munich, Technische Universität München, Munich, Germany; ^2^ Division of Congenital and Pediatric Heart Surgery, University Hospital of Munich, Ludwig-Maximilians-Universität, Munich, Germany; ^3^ ARTORG Center for Biomedical Engineering Research, University of Bern, Bern, Switzerland; ^4^ Department of Biomedical Research, University of Bern, Bern, Switzerland; ^5^ Department of Cardiac Surgery, Ludwig-Maximilian University, Munich, Germany; ^6^ Department of Cardiac Surgery, University Hospital, Zürich, Switzerland

**Keywords:** biomedical engineering, patch, congenital, biogenic polymers, innovation

## Abstract

**Objective:**

Currently used patch materials in congenital cardiac surgery do not grow, renew, or remodel. Patch calcification occurs more rapidly in pediatric patients eventually leading to reoperations. Bacterial cellulose (BC) as a biogenic polymer offers high tensile strength, biocompatibility, and hemocompatibility. Thus, we further investigated the biomechanical properties of BC for use as patch material.

**Methods:**

The BC-producing bacteria *Acetobacter xylinum* were cultured in different environments to investigate optimal culturing conditions. For mechanical characterization, an established method of inflation for biaxial testing was used. The applied static pressure and deflection height of the BC patch were measured. Furthermore, a displacement and strain distribution analysis was performed and compared to a standard xenograft pericardial patch.

**Results:**

The examination of the culturing conditions revealed that the BC became homogenous and stable when cultivated at 29°C, 60% oxygen concentration, and culturing medium exchange every third day for a total culturing period of 12 days. The estimated elastic modulus of the BC patches ranged from 200 to 530 MPa compared to 230 MPa for the pericardial patch. The strain distributions, calculated from preloaded (2 mmHg) to 80 mmHg inflation, show BC patch strains ranging between 0.6% and 4%, which was comparable to the pericardial patch. However, the pressure at rupture and peak deflection height varied greatly, ranging from 67 to around 200 mmHg and 0.96 to 5.28 mm, respectively. The same patch thickness does not automatically result in the same material properties indicating that the manufacturing conditions have a significant impact on durability

**Conclusions:**

BC patches can achieve comparable results to pericardial patches in terms of strain behavior as well as in the maximum applied pressure that can be withstood without rupture. Bacterial cellulose patches could be a promising material worth further research.

## VISUAL ABSTRACT

**Key question:**

Can bacterial cellulose patches offer comparable mechanical properties as conventional patch materials?

**Key findings:**

When compared to bovine pericardial patches, BC patches produced comparable strain distribution maps and inflation behavior.

**Take-home message:**

The study showed that bacterial cellulose is a promising biomaterial for the application as patch material in congenital cardiac surgery.

**Central image legend:**

train distribution map analysis in horizontal direction and vertical directions for representative BC patch.

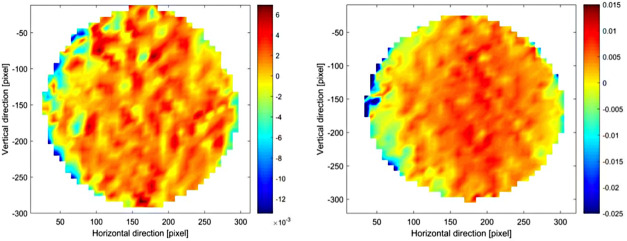

## Introduction

In the United States, nearly 1% of all newborn babies suffer from a congenital heart defect, which is the most common malformation at birth. About one of four infants is diagnosed with a critical defect and will need surgery within the first year of life (1). Pediatric cardiac surgery has achieved numerous significant advancements in the last few decades (2). Some improvements are due to the implementation of new materials that make it possible to correct even anatomically complex cases (3). But as more patients are surviving their interventions, focus has shifted from survival to the long-term quality of life of these former pediatric patients. With regard to this issue, one of the most critical problems is the limited availability of high-performing devices and materials for this category of patients. As a result, currently used materials may help save lives, but they do not offer unrestricted lifespans (2, 3). In addition to already known phenomena of tissue degeneration, there are additional concerns with foreign materials in the pediatric population. The use of biological patch materials such as bovine pericardium has limitations since these fail to grow, renew, or remodel. Calcification of prosthetic patches even occurs earlier and progresses faster than in adults (4). Moreover, current materials are prone to infection, immunologic reactivity and thrombosis eventually leading to reoperations (3). Therefore, it becomes evident that there is a huge potential for improvement. To date, the optimal material for use in pediatric cardiac surgery has not been identified; not only are there no optimal materials available but there are no optimized materials either (5).

A promising concept in material developments are polymer derivatives as they offer optimal hemodynamic and improved durability, while avoiding a complicated manufacturing process as they are not derived from animal tissue. Current technological advances in the production of polymer-made patches are an interesting and viable option: they can be easily molded to the desired shape and closely resemble the architecture of the natural heart. Currently, the primary concern is identifying a source material with adequate durability and low thrombogenicity (3).

Bacterial cellulose (BC) is a biogenic polymer produced by bacterial strain *Acetobacter xylinum (A. xylinum)* and has the potential to overcome these difficulties when used as patch material in congenital cardiac surgery. Previous studies could demonstrate that there was neither acute nor chronic inflammation; on the contrary, BC grafts became even vascularized and contained newly synthesized collagen as well as endothelial cells attached to the cellulose scaffold as it resembled a basal membrane (6–8). BC exhibits high tensile strength and biocompatibility with mechanical characteristics that make it a promising component for tissue engineering (9–11).

This study aimed to investigate the biomechanical properties of bacterial cellulose as a material for congenital heart surgery. *A. xylinum* was cultured to produce BC patches (BCP). In comparison to commercially available xenograft material, the mechanical and morphological properties of biogenic polymer material were analyzed.

## Materials and methods

### Bacterial strain

For all experiments, *Gluconacetobacter xylinus* subsp. sucrofermentans BPR 2001 (JCM 9730) from the American Type Culture Collection (ATCC) was used. The strain was stored in 2 ml Eppendorf tubes in a 20% glycerol solution at −80°C until use. If not stated differently, the used scaffolds for culturing *A. xylinum* were Petri dishes with a diameter of 50 mm (Cell Culture Dish 60 mm × 15 mm Style, treated, non-pyrogenic polystyrene, sterile, from Signa-Aldrich USA, St. Louis, MO, United States). To control the metabolic activity of *A. xylinum*, the pH of the culturing medium was measured (SevenEsay pH Meter S20, Mettler-Toledo AG, Analytical, Schwerzenbach, Switzerland) every time the medium was exchanged, and the experiment was finished.

*Name and identification number:* Gluconacetobacter xylinus (ATCC ®700178TM). *Designation:* JCM 9730 [BPR2001, FERMBP 4545, LMG 18788].

*Deposited Name: Acetobacter xylinus* subsp. sucrofermentans Toyosaki et al. *Product Description:* Deposited as and referred to as the type strain of *Acetobacter xylinus* subsp. sucrofermentans; produces large amounts of cellulose.

### Inoculum prepreparation

The *A. xylinum* were stored in Eppendorf tubes (2 ml) in a 20% glycerol solution at −80°C until use. To ensure good quality of the starting colony, the frozen bacteria had to be cultured on an Agar plate. The Agar plate ensured that the thawed bacteria were exposed to a high amount of nutrients, which led to an accelerated growth. The medium used to prepare the Agar plate was the one recommended by the Biosource Center, which delivered the bacteria. It consists of glucose (C6 H12 O6, Sigma-Aldrich Chemie GmbH, Steinheim, Germany), yeast extract (GIBOCOBRL, Life Technologies, Paisley, Scotland), calcium carbonate (CaCO_3_, Sigma-Aldrich Chemie GmbH, Steinheim, Germany), Agar (Microbiology Agar-Agar, Dr. GROGG CHEMIE AG, Darmstadt, Germany), and distilled water from our lab. For the exact formulation and the autoclaving instructions, see Supplementary Table S1. After autoclaving, the medium (ATCC Medium 459) was placed in a water bath to be cooled down to approximately 30−40°C, and molded in a Petri Dish with a diameter of 90 mm. As soon as the medium is solidified, the agar plate is ready for use. One of the Eppendorf tubes with the frozen bacteria/glycerol solution is thawed and divided into two agar plates and spread over each of them, in parallel to the incubation of the Agar plates, the culture medium (medium with 2.381% glucose, for cellulose production). Supplementary Table S2 shows the chemical formula, corresponding name, and the percentage share of all medium components. All components were ordered from Sigma-Aldrich Chemie GmbH, Steinheim, Germany.

**Figure 1:**
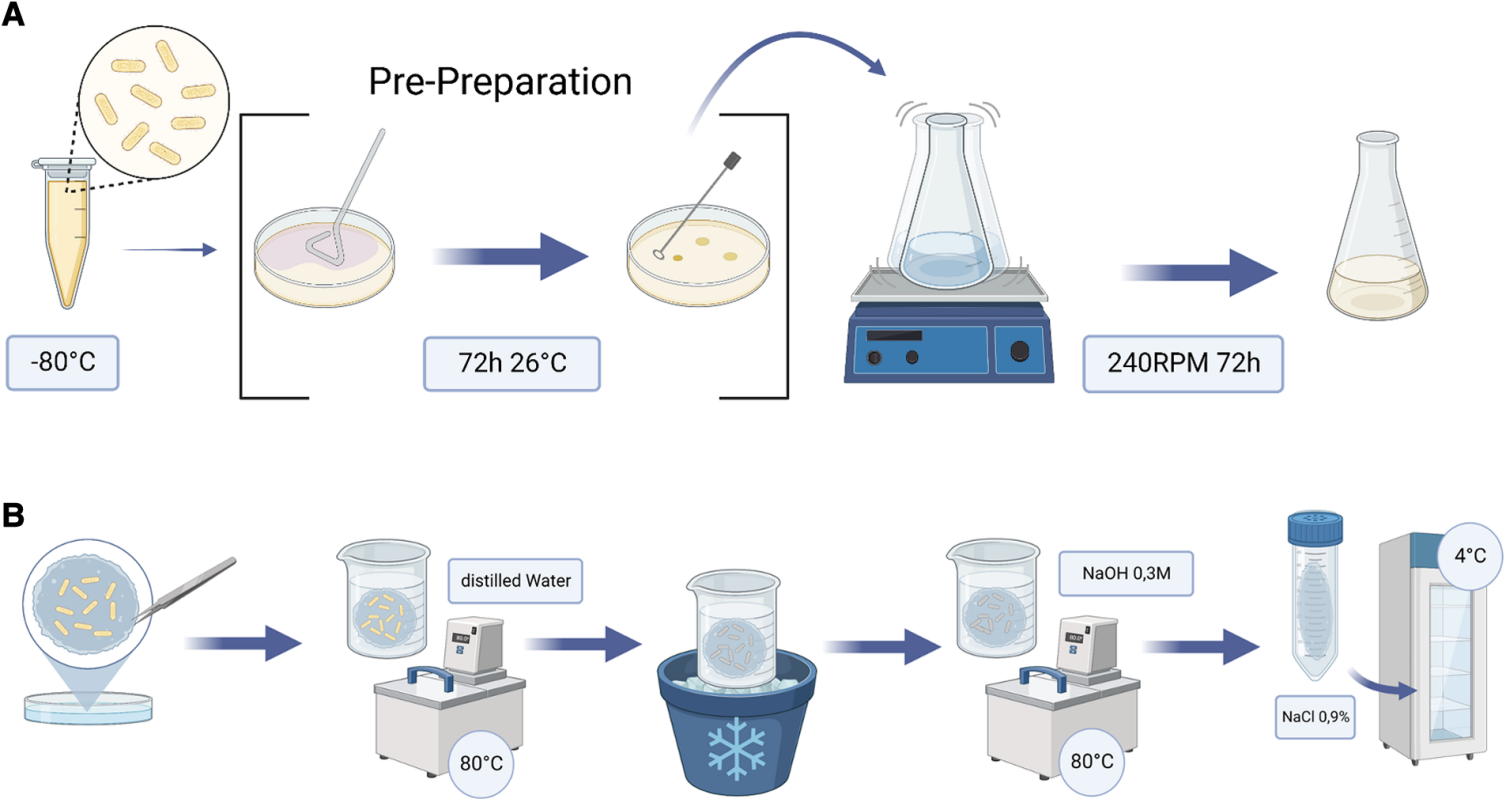
Flow charts of the steps undertaken to produce BC patches. (**A**) illustrates the extra step of prepreparation. (**B**) shows the postpreparation each patch underwent after the growing period. BC, bacterial cellulose.

After 72 h incubation at 26°C, a single colony was picked and dissolved in 200 ml Cellulose Production Medium (Supplementary Table S2) and placed in a 500 ml Erlenmeyer Flask on the shaker at 240 RPM for 72 h. The bacteria in the medium, now termed inoculum, had sufficiently multiplied by then and were ready to use (Figure 1A). It was then added in the Petri dishes to the Cellulose Production Medium at a ratio of 1:10.

### Bioreactor setup

A bioreactor was built to control temperature, oxygen, and nutrient delivery, as well as waste gas removal, to ensure a sterile environment for growing and repeatable patches. To be able to easily sterilize the production area, we used a retractable incubation box in a water bath with a preinstalled heater to keep the temperature at 29°C + −0.5°C (Supplementary Figure S1).

### Postpreparation

**Figure 2:**
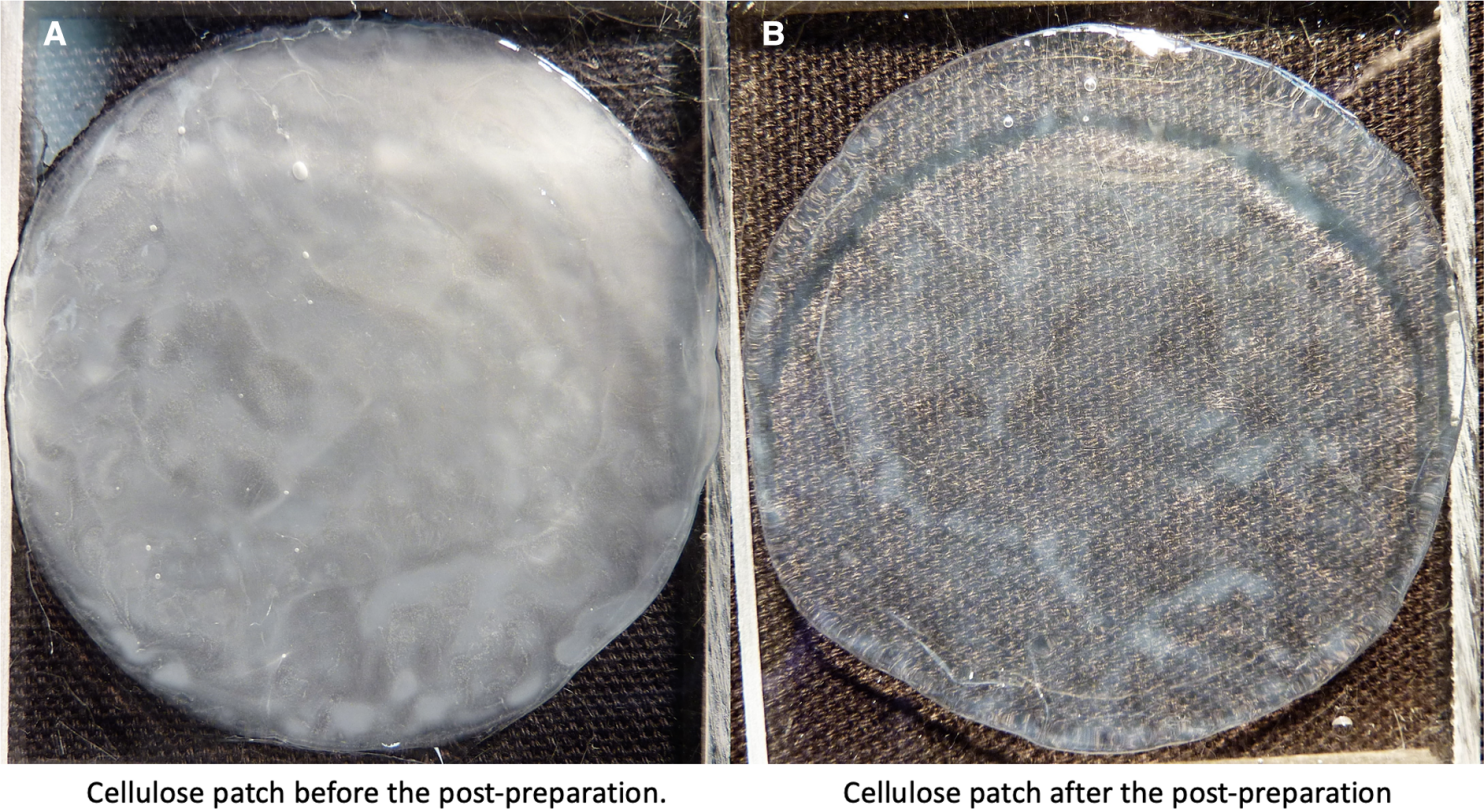
BC patch incubated 6 days at 29°C. (**A**) shows the patch before the postpreparation. (**B**) shows the same patch after the post preparation. BC, bacterial cellulose.

After culturing, the obtained membranes were boiled in distilled water at 80°C for 30 min. Subsequently, the patches were cooled down before boiled again at the exact same temperature and time in a 0.3M sodium hydroxide (NaOH) solution (Sigma-Aldrich Chemie GmbH, Steinheim, Germany) to clear out all bacteria. After another cooling phase in distilled water, the patches were finally stored in 0.9% NaCl solution at +4°C in the fridge (Figures 1B, 2).

### Data processing and analysis

The data analysis was conducted using MATLAB (MathWorks, Natick, MA, United States). The calculation of the strain distribution on the BC patch was done using particle image velocimetry. For the detection of the deflection height, a custom made MATLAB script was used. The analysis could be subdivided in four major sections:

(1) Calculation of mapping function to correct distortions of the images.
(2) Image preprocessing to enable a better contrast of the images (separate algorithms for strain and deflection height analysis).
(3) Analysis of strain distribution on BC patch (top view of BC patch).
(4) Analysis of deflection height of BC patch (side view of BC patch).

Further details these four major sections are described in the Supplementary material.

### Mechanical testing

For the purpose of mechanical testing four, representative BC patch samples were analyzed. The baseline in all experiments was a state-of-the-art pericardium patch (SJM™ pericardial patch) identically measured. The BCPs used were: BCP-1 (12 days incubation in ambient conditions), BCP-2 (12 days incubation in Bioreactor Setup), BCP-3 (6 days incubation in Bioreactor Setup), BCP-4 (12 days incubation in Bioreactor Setup), and the pericardium patch.

To gain further insight into the mechanical properties of the BC patches, we designed an inflation test-stand: each sample was fixed by a clamping ring and placed in a cylinder, which could then be connected to a water column. The patch was then inflated until rupture by the applied water pressure. Pictures of the process were obtained by a camera and the pressure sensor at the bottom of the column recorded the applied pressure (Supplementary Figure S2). With this setup, which is similar to the one presented by Buerzle et al. and Chanliaud et al (12, 13), a maximum pressure of 200 mmHg could be detected. As soon as the patch ruptured, the recording was stopped. Parameters analyzed were pressure at rupture and maximum deflection height while inflating. The elastic modulus (E) was estimated using the following relation between pressure (P) and deflection height (h) for a circular membrane (14).

(1) $$P=\frac{4\cdot \sigma_{0}\cdot t}{a^{2}}\cdot h+\frac{8\cdot E\cdot t}{3\cdot a^{4}(1-\upsilon )}\cdot h^{3}$$

where $\sigma_{0}$ is the residual stress of the membrane, *t* is the membrane thickness, *a* is the membrane radius, and υ is the Poisson ratio. The BC was assumed to be incompressible (υ=0.5). The first part of the measured pressure-deflection curves were fitted to $P=a\cdot h+b\cdot h^{3}$ using the least squares method in MATLAB, and the elastic modulus was extrapolated from coefficient *b* using Equation 1.

### Detection of deflection height

The Images obtained from the patches in sideview were preprocessed using different MATLAB functions. This simplified the following edge detection by the canny filter allowing it to be plotted into a new vector (Supplementary Figure S2). The value of this was then applied to a Polynomial-Function by MATLAB for the curve fitting. In a last image, only the fitted curve was plotted (Supplementary Figure S2). The maximum deflection of the BC patch was calculated by subtracting the maximum y-value of the preloaded and the maximum y-value of the BC patch, just before rupture.

### Particle image velocimetry and strain distribution images

For this method, tracer particles were placed on top of the clamped patch before adding pressure to the mechanical testing setup. A mirror was mounted on top of the clamping ring to be able to detect top view images. Hence, we were able to track each particle in its movement during the inflation resulting in a movement vector. The observed motion of the BC patches was very small, and the general strain distribution behavior (computed as the spatial gradient of the displacement) of the BC patches were of interest (Supplementary Figure S3). Therefore, only two preprocessed frames were loaded into the particle image velocimetry (PIV) software. The first frame was the one at time point 0, where the BC patch was preloaded. The second frame loaded into the PIV software was the frame at *p* = 80 mmHg. A pressure of 80 mmHg was chosen to enable the analysis of all four chosen representative BC patches. We could then create a display of the movement of the particles and therefore the patch carrying the particles between these two timepoints. The PIV program used for this was a MATLAB based open-source tool named PIVlab—Time-Resolved Digital Particle Image Velocimetry Tool (for more details about the tool, all its features, and for downloading the software, see http://pivlab.blogspot.ch/). To visualize the strain distribution on the BC patch, a p-color plot in MATLAB was plotted.

## Results

### Examination of growing conditions

**Figure 3:**
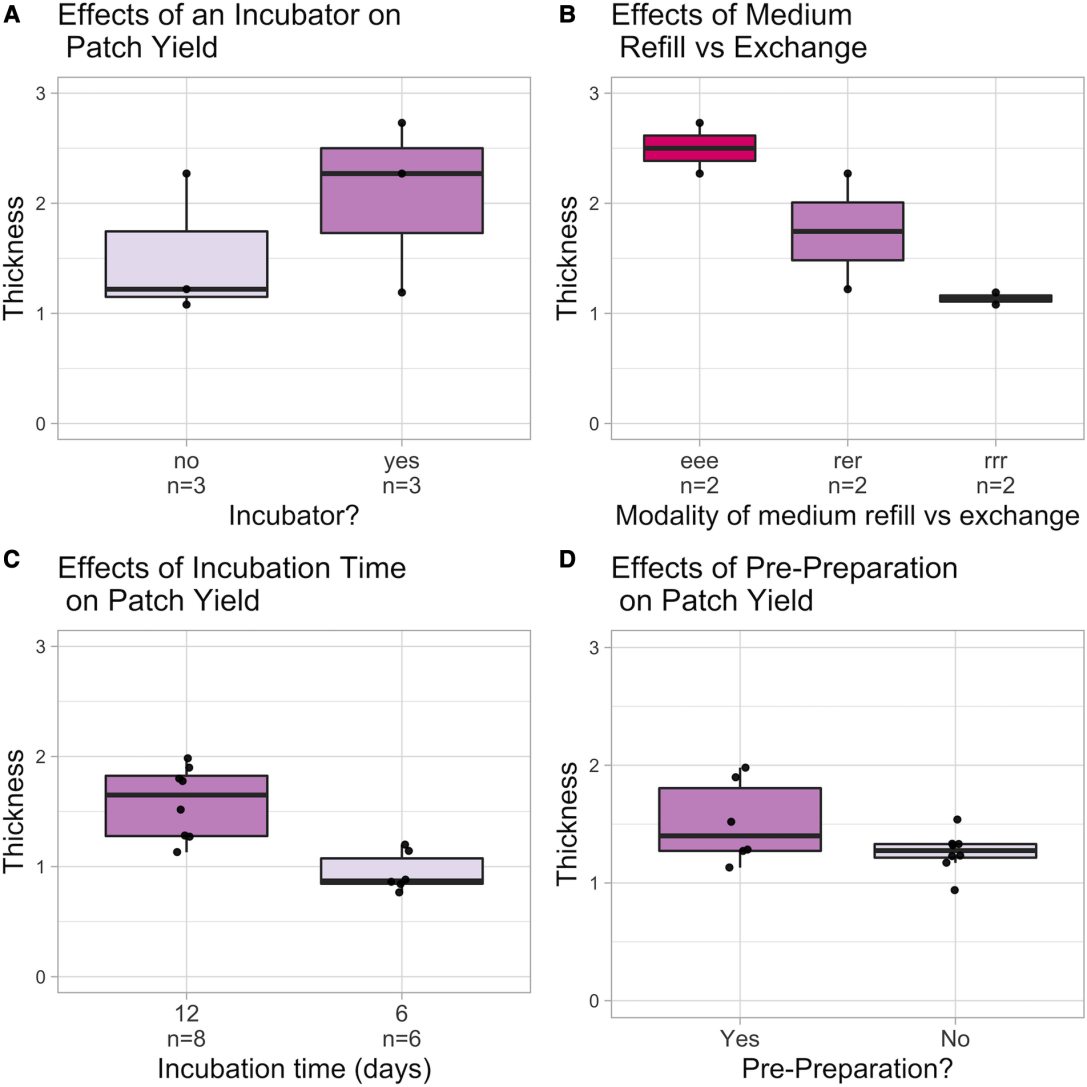
Overview of the investigated culturing conditions. (**A**) examines the use of an incubator: (**B**) compares different medium exchange and refill schemes. (**C**) depicts the influence of growth time. (**D**) depicts the influence of prepreparation. Thickness is in mm.

The optimal culturing conditions for the BC-producing bacteria strain *A. xylinum* were analyzed in a pilot study. It is assumed that the control of temperature, O_2_, and nutrient supply as well as the removal of the waste gas by a bioreactor would result in higher BC yield. Therefore, patches grown at ambient conditions with BC grown in the controlled setup of a bioreactor were compared (Figure 3A). To gain further insight into how nutrient supply and medium exchange influence the thickness of the grown BC, different refill and exchange schemes were examined: over the growth period of 12 days, the Petri dishes were either subjected to three exchanges of medium after days 3, 6, and 9 (eee) or refills at days 3 and 9 with one exchange at day 6 (ere) and 3 refills at days 3, 6, and 9 (rrr), respectively (Figure 3B). In addition, different growth times were evaluated to see whether the bacteria may stop growing at a certain point, even if sufficient nutrients were still available due to medium changes (Figure 3C). Figure 3D demonstrates that prepreparing the inoculum by giving the bacteria time to multiply on a solid agar plate and picking only on colony before putting them in the shaker had a strong influence on the yielded thickness of the BC membranes. Thus, for all further experiments, pre-prepared bacteria in the Bioreactor with Medium Refills every 3 days were used.

### Mechanical testing setup—comparison of maximum withstood pressure

**Figure 4:**
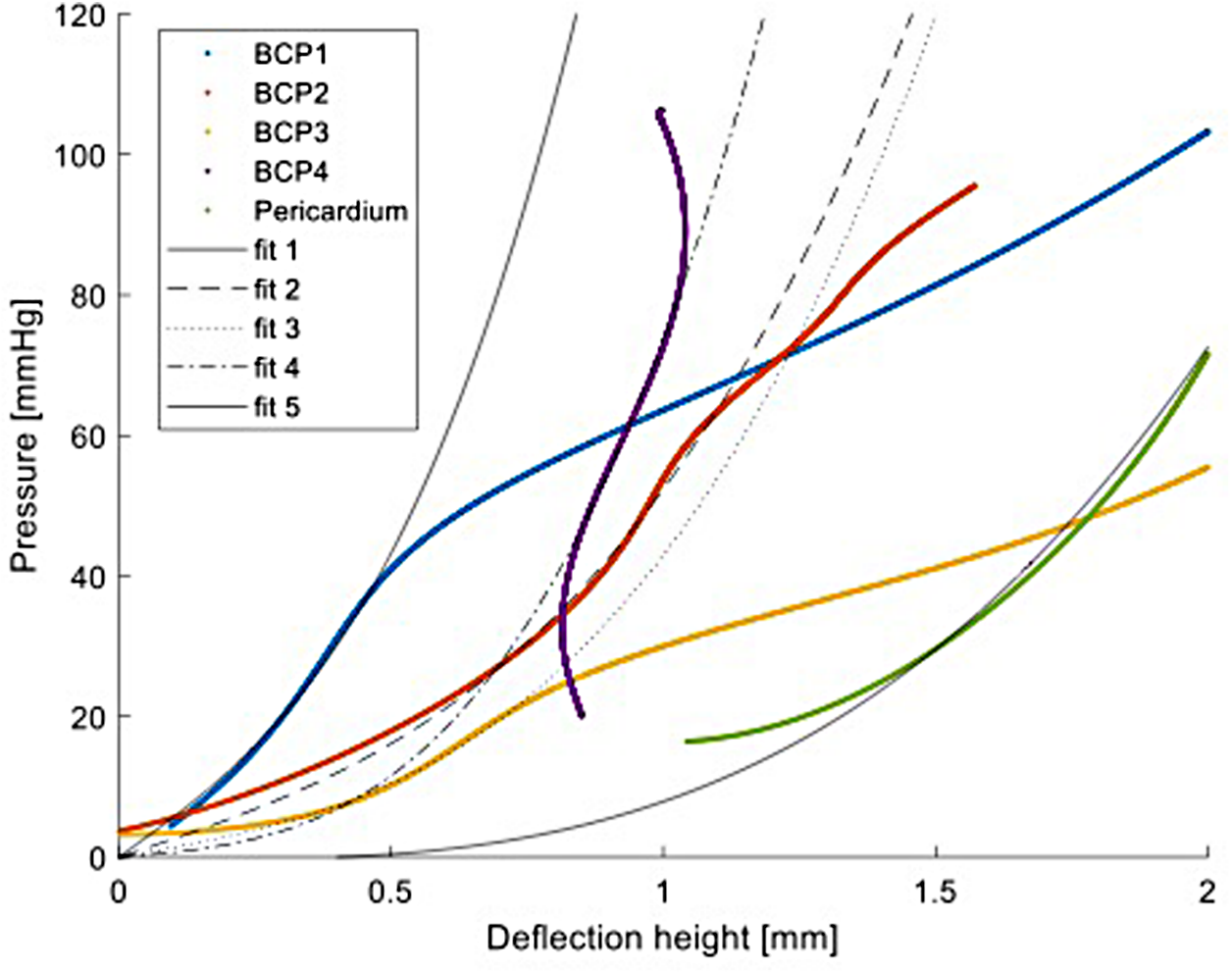
Pressure vs. deflection height for all four BC patches (BCP1-4) and the pericardium patch. Fit 1–4 and fit 5 are the least squares method fits to estimate the elastic modulus of BC1–4 patches and the pericardium patch, respectively, from Equation 1. BC, bacterial cellulose.

Figure 4 shows the applied pressure against the deflection height for all four BC patches and the pericardium patch up to approximately 100 mmHg and a deflection height of 2 mm (Supplementary Figures S5–S9 show the applied pressure and the deflection height over time for BCP-1-4 and for the pericardium patch). Due to the insufficient image quality, only the first values are missing for patch BCP-4. For the pericardium patch, the first part is also missing. Figure 4 shows that the slopes of the different patches differ from each other and changes behavior with increasing pressure. The elastic modulus was estimated to be 530 MPa for BCP-1, 200 MPa for BCP-2, 340 MPa for BCP-3, and 330 MPa for BCP-4, which was comparable to the value estimated for the pericardium patch which was 230 MPa (see fitted curves in Figure 4). After the initial part of the inflation testing, BCP-1–3 show larger increase in deflection heights leading into rupture at 151, 100, and 82 mmHg, respectively. BCP-4 and the pericardium patch, however, did not rupture within the maximum applied pressure of 200 mmHg.

Furthermore, as discussed in Cacopardo et al. (15), the measured elastic modulus corresponds to an apparent elastic modulus. The mechanical properties of BC are likely viscoelastic in nature, which means that static load conditions are expected to result in permanent deformation. In cyclic loading conditions, the apparent elastic modulus is expected to increase for higher frequencies. In the present experiments, no permanent deformations were observed (except just before material failure). The patches, on the other hand, will be more precisely analyzed using dynamic measurement, as the current measurement cannot provide any information about the patches’ long-term durability. A high cycler capable of taking measurements over several weeks is an option for this purpose.

### In plane displacement

**Figure 5:**
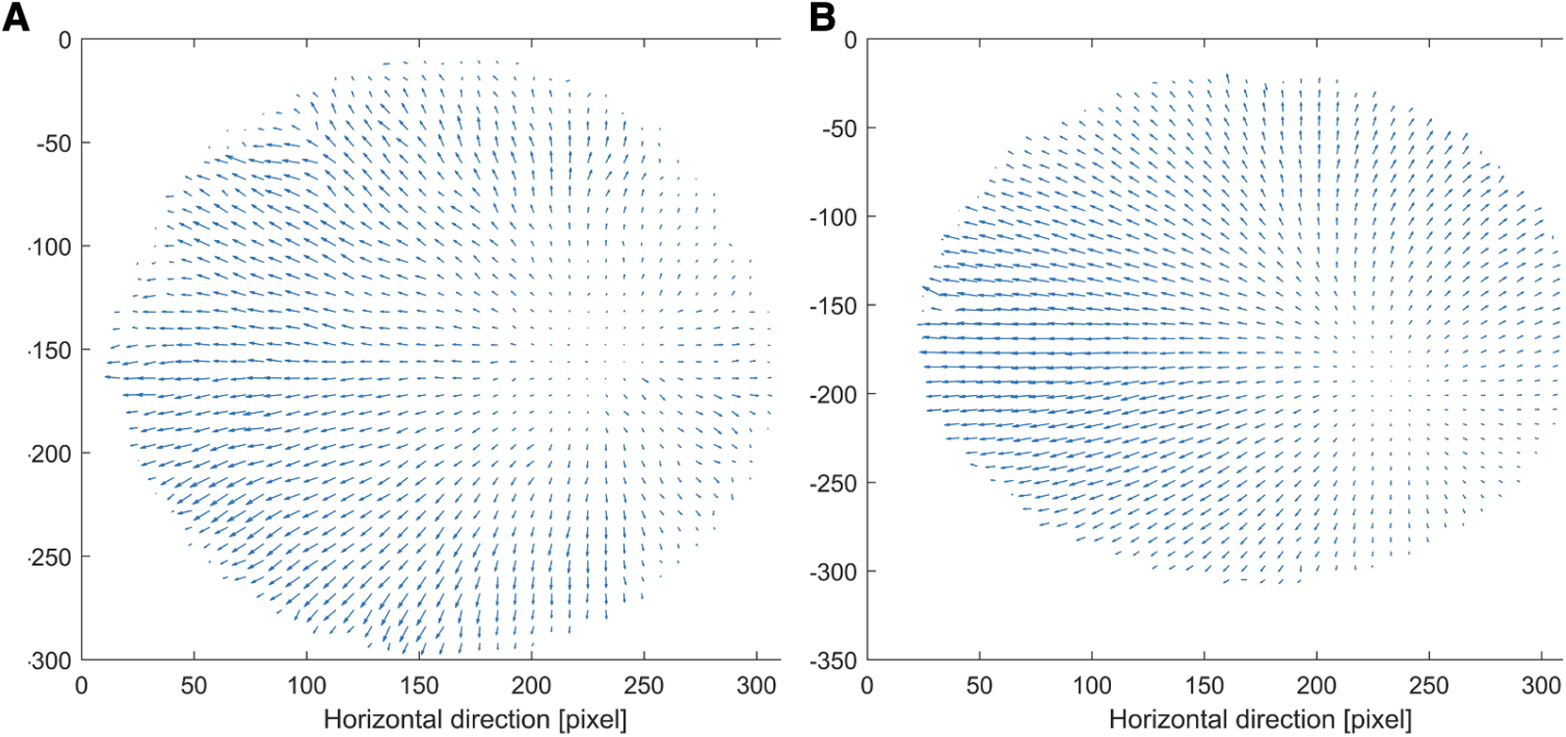
In plane displacements of BCP-4 (**A**) and the pericardium patch (**B**). The unit of vectors is mm and the axis are in pixel with respect to the tailored image. BCP, bacterial cellulose patch.

The BC patches and the pericardium patch show an overall similar displacement behavior. Figure 5 shows the displacement in plane for BCP-4 (Figure 5A) and the pericardium patch (Figure 5B), calculated for the displacement of the patches from the first frame, which corresponds to the preloaded state and to the frame corresponding to an applied pressure of 80 mmHg. A pressure of 80 mmHg was chosen to ensure that all patches could be compared and none of them was already ruptured. All patches showed a nice vector field with noisy signals only at the edge of the patch. Moreover, all patches had their zero-shift point in approximately the same area. The zero-shift corresponds to the point in which the camera is exactly perpendicular to the patch. For all patches, there occur shifts in the horizontal and vertical directions. All patches show in their middle a clear shift in the horizontal direction to the left. Supplementary Figure S4 exhibits a clear shift to the left at the top boarder for BCP-1, 2, and 3. The whole patch did not stretch equally in all directions but moved to the left. The BCP-4 patch and the pericardium patch show a homogeneous distribution of the shifts in all four directions.

### Plane strain distribution analysis

**Figure 6:**
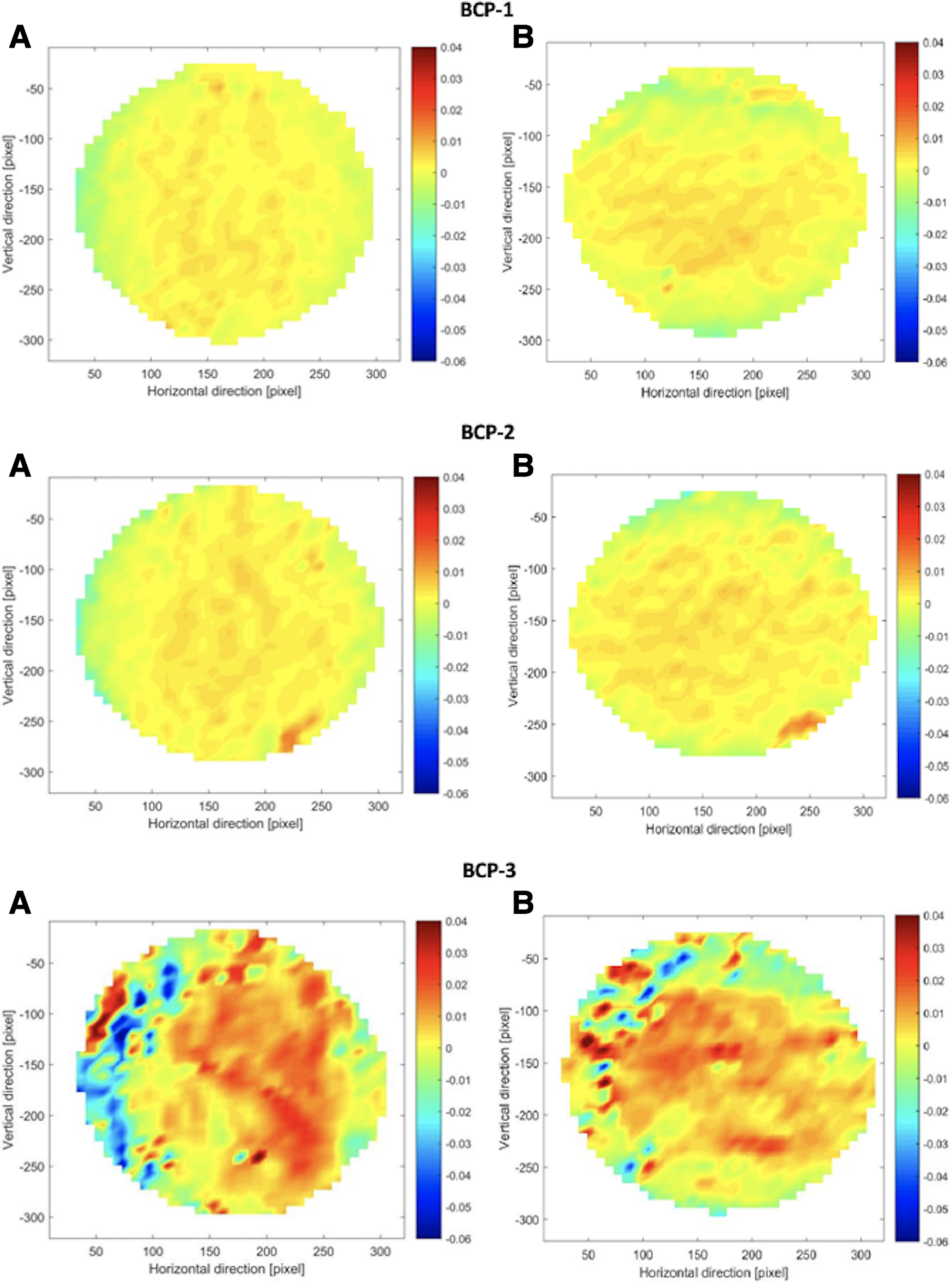
Strain distribution maps for patch BCP1, 2, and 3. For each patch, all (**A**) show the strain in the horizontal direction and all (**B**) show the strain in the vertical direction. The strain map is calculated from the preloaded state (approximately *p* = 2 mmHg) until a pressure of 80 mmHg. BCP, bacterial cellulose patch.

**Figure 7:**
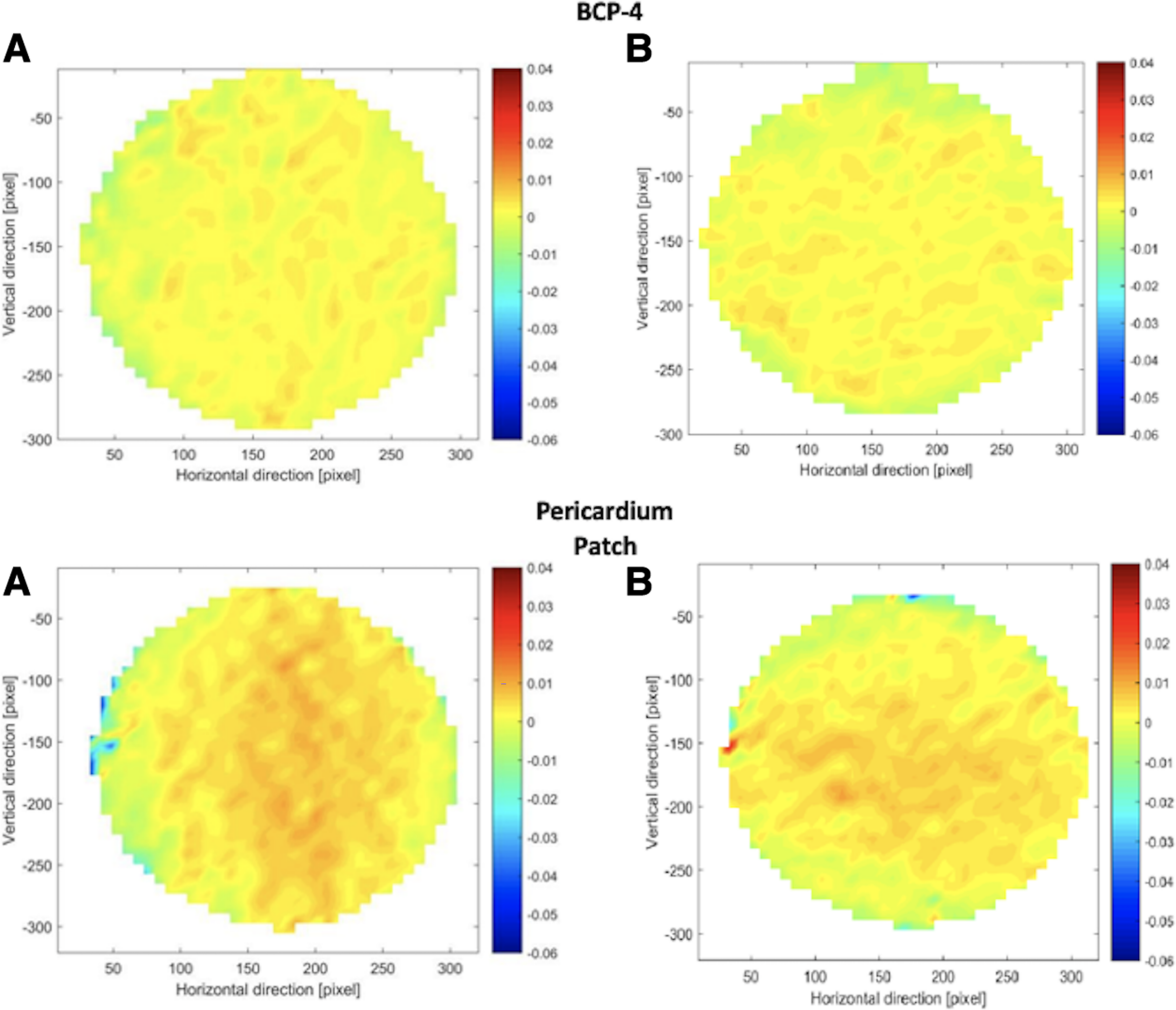
Strain distribution maps for BCP-4 and pericardium patch. For each patch, all (**A**) show the strain in the horizontal direction and all (**B**) show the strain in the vertical direction. The strain map is calculated from the preloaded state (approximately *p* = 2 mmHg) until a pressure of 80 mmHg. BCP, bacterial cellulose patch.

Plane Strain Distribution Maps, which plot Particle Image Velocimetry data, were created to visualize how forces operate during inflation testing. The strain distribution maps for all BC patches, as well as for the pericardium patch, were similar. Figures 6, 7 show the strain distribution maps for all the BC patches and the pericardium patch. For each patch, both the normal strain in the horizontal direction and the normal strain in the vertical direction are shown [(A) and (B), respectively]. The strain is calculated from the preloaded state (approximately *p* = 2 mmHg) until a pressure of 80 mmHg is reached. Figures 6, 7 show the comparison of all patches with the same color coding. The results reveal that the strains occurring in patch BCP-3 are at least 62% higher compared to all other patches and the pericardium patch. The findings show that the strains observed in patch BCP-4 are at least 10 times smaller than the strains found in all other patches, including the pericardium patch. The largest strains occur at the center of the patch and gradually decrease toward the border. Nonetheless, the blue parts at the border indicate compression. This behavior can be explained by considering only the plain strains and disregarding the deflection height. Surprisingly, the strain distribution map for the three BC patches that ruptured (BCP-1, BCP-2, and BCP-3) shows high strains in the area of the patch failures. Patch BCP-2 fails in the bottom right corner, precisely at the point of greatest strain, whereas the other two patches fail in the patch's center.

## Discussion

A majority of congenital defects require either intracardiac reconstruction or repair of pulmonary and/or systemic inflow or outflow. To close intracardiac shunts such as ventricular septal defect (VSD) or reconstruct outflow tract or great vessels, pericardium, polyester, or Polytetrafluoroethylene (PTFE) can be used. Current materials have limitations and thromboembolism, infection, shrinkage, calcification, and tissue overgrowth may appear following use of synthetic materials (3).

To date, no optimal material for use in pediatric cardiac surgery has been identified; only optimized materials are available (16).

Prospective randomized trials on a large scale and systemic histopathological workups are preferred. In the future, new emerging techniques such as 3D printing, computational modeling, and tissue engineering may aid in the provision of personalized treatment options with optimal geometrical and flow properties (17).

Materials with comparable elasticity to cardiac tissue and increased biocompatibility, allowing for early neo-endothelialization and resulting in a decreased inflammatory response, are welcome. Nevertheless, only a few research studies evaluating different patch materials have been performed in large animal models so far (11, 18).

BC is a biomaterial that potentially meets several of the parameters listed above. It is a hydrogel made up of a nanofiber network with fiber sizes ranging up to 100 nm, which contains 99% water (11, 19). Furthermore, Bacterial nanocellulose (BNC) is structurally similar to collagen assembly, which may provide a better substrate for tissue ingrowth and endothelialization. Furthermore, numerous studies have shown that BNC has excellent biocompatibility with minimal local inflammatory reaction (6, 7, 10, 11, 20).

In a study by Lang et al, BC material was evaluated as a path material for transcatheter VSD closure and *in vivo* biocompatibility was assessed in a chronic pig model. The group concluded that BC patches may be used for closure of VSD with good mid-term results and that their biocompatibility was satisfactory (11) However, data on elasticity as well as the testing of various BC modifications of this new patch material, such as different forms of BC in comparison to other patch materials, are still lacking.

The objective of the present project was to investigate further whether BC is a biomaterial with suitable mechanical properties to be used as patch material in comparison to commercially available xenogenic material. Thus, we created a bioreactor for controlling the culture parameters and analyzed the optimal culture conditions for *A. xylinum* bacteria to produce BC. In addition, the resulted BC patches were characterized in a mechanical testing setup employing various analyses for comprehensive material characterization.

### Evaluation of BC growth

Even though the bacteria would still grow in ambient conditions, it shows that more parameters influencing the yield could be controlled by using a bioreactor: temperature, O_2_ supply, and nutrients as well as removal of the waste gas were monitored in this setup. Furthermore, the retractable incubation box could easily be sterilized to ensure that the experiments do not influence each other. Milli-Q water, used to fill the water tank, guaranteed that no contamination of the water occurred over the whole experiment. It is widely agreed that *A. xylinum* are obligate aerobe bacteria and, therefore, oxygen is a major component of their metabolism (21). However, there are various O_2_ concentrations used, and it has even been shown that oxygen supply is not the limiting factor regarding BC yield over longer cultivation periods (22). This was observable to some extent, as patches continued to grow in ambient conditions despite the fact that this setup was susceptible to numerous uncontrollable variables. As the best results were obtained when the medium was exchanged rather than simply refilled, we hypothesized that it is more important to control both the supply of nutrients and the removal of waste products than to simply provide new nutrients. This was demonstrated by the fact that the thickness of the patches from the exchange setup (eee) was greater than the thickness of the patches from the refill or one exchange setups (rer, rrr). The number of patches produced per group was limited, but as the number of patches increases, this trend will undoubtedly become significant. We attribute great importance to keeping the pH value at a level between 4.0 and 6.0, as this is described to be the optimum range for *A. xylinum* to produce durable BC (23). As we were not able—even with the exchange setup (**eee**)—to keep the pH at a constant level, we will therefore install a pumping device beneath the growing patch at the surface in future experiments and by that also maintain constant glucose supply.

### Mechanical testing setup—comparison of maximum withstood pressure

During mechanical testing, maximum inflation of the patches as well as maximum withstood pressure were recorded to investigate a possible relation between the thickness of the sample and those parameters. The maximum pressure withstood was visibly influenced by the thickness of the sample. Interestingly, the duration of culture and thus also the absolute number of medium changes seems to influence the resistance of the material reflected (Figure 3). This effect is even more apparent when comparing the maximum withstood pressures of all samples: only BCP-4 patches were capable to keep up with the baseline pericardium patch and did not rupture during the entire experiment (Figure 3). In future experiments, the effects of preprocessing on the bacteria should be investigated more in detail: it is conceivable, for example, that the proportion of active living bacteria is higher than without preprocessing and that the available nutrients can therefore be linked to more cellulose. This could be accomplished, for instance, by more precisely analyzing the exchanged medium to determine how much glucose is consumed as an indicator of the activity of the bacteria. Furthermore, as discussed in the study by Cacopardo et al. (15), the measured elastic modulus corresponds to an apparent elastic modulus. Furthermore, the mechanical properties of BC are likely viscoelastic in nature, which means that static load conditions are expected to result in permanent deformation while cyclic load conditions during a physiological heartbeat are likely fast enough that viscoelasticity does not dominate. No permanent deformations were observed in our experimental testing (except just before material failure). The patches, on the other hand, will be more precisely analyzed using dynamic measurement, as the current measurement cannot provide any information about the patches’ long-term durability. A high cycler capable of taking measurements over several weeks is an option for this purpose.

### Outlook

Fabrication of composite scaffolds may be a potential method to enhance the stability of novel implant materials. Clinical use of decellularized tissues is already widespread, but their biological variability makes them challenging to process. With electrospinning (ESP) technology, it may be possible to process synthetic and biogenic scaffolds (24). ESP has been a standard technique for creating scaffolds in tissue engineering, permitting the combination of different materials and the modification of fiber diameters, fiber orientations, and porosities (25). In addition, a variety of postprocessing techniques allows for optimized tissue remodeling, enhanced hemocompatibility, and controllable biodegradation of electrospun scaffolds (25–28). Extensive testing of the material's properties should be followed by an examination of its viscoelastic properties, which necessitates the use of more sophisticated testing techniques than those typically employed (15, 29).

## Conclusion

The mechanical characterization of the BC patches revealed that BC is a homogeneous, stiff material with elastic modulus between 200 and 530 MPa and strain values comparable to a bovine pericardium patch. The displacement patterns, as well as the strain maps, are similar for all tested BC patches, which indicated that the mechanical behavior is reproducible. The cellulose patch, which was assumed to have a dense mesh, inflated less with increasing pressure compared to the pericardium patch. From this, we assume that BC patches with a dense cellulose fiber mesh are stiffer (and therefore more resistant to pressure?) than the bovine pericardium.

## Data Availability

The original contributions presented in the study are included in the article/**Supplementary Material**, further inquiries can be directed to the corresponding author.

## Supplementary material

SUPPLEMENTARY FIGURE 1

Schematic overview of the final setup of the bioreactor and the connection of all parts. First, oxygen and pressurized air were connected to the Y-branch, and both pressures were set to 1bar. Second, the outflow tract of the Y-tract was connected to an occlusion element to reduce the gas flow rate. Third, the supply gas was led from the occlusion element directly into the incubation box. The supply gas tube ended in a water reservoir, where the gas was humidified. The Petri Dishes with the A. Xylinum seeded in the culture medium and were placed in the incubation box next to the water reservoir, and the lid was closed. The closed incubation box with gas supply was placed in the glass tank, which was heated to a temperature between 29°C and 30°C. The lid of the glass tank was closed, and the isolation mat was placed around it.

SUPPLEMENTARY FIGURE 2

Schematic overview (a) Water column inflates BC sample. Disposable cylinder pressure sensor. 25Hz camera takes photos. Dual lighting improves image contrast. (b) Cylinder with two BC patches. Black shows the preloaded BC patch. Preloaded vs. maximally deflected BC patch determines deflection height.

SUPPLEMENTARY FIGURE 3

Schematic overview of the image pre-processing steps of the top view with the applied function in each step and the resulting image.

SUPPLEMENTARY FIGURE 4

In plane displacements of BCP-1, 2 and 3. The unit of the vectors is mm and the axis are in pixel with respect to the tailored image.

SUPPLEMENTARY FIGURE 5

Pressure and deflection height over time for BCP-1.

SUPPLEMENTARY FIGURE 6

Pressure and deflection height over time for BCP-2.

SUPPLEMENTARY FIGURE 7

Pressure and deflection height over time for BCP-3.

SUPPLEMENTARY FIGURE 8

Pressure and deflection height over time for BCP-4.

SUPPLEMENTARY FIGURE 9

Pressure and deflection height over time for the pericardium patch.
