# Supplementary material for: Biogenic polymer-based patches for congenital cardiac surgery: a feasibility study
Source: Front Cardiovasc Med. 2023 Jun 22;10:1164285. doi: 10.3389/fcvm.2023.1164285 (PMC10325621; doi:10.3389/fcvm.2023.1164285)
Supplement: Supplementary file 1 [file Table1.docx]

Supplemental Table 1: ATCC Medium 459

| Glucose | 50.0 g |
| --- | --- |
| Yeast Extract | 5.0 g |
| CaCo_3_ | 12.5 g |
| Agar | 15.0 g |
| Distilled Water | 1.0 L |

Supplemental Table 2: Cellulose Production Medium

| KH_2_PO_4_ | 0,7% |
| --- | --- |
| MgSO_4_ x 7 H_2_O | 0,213% |
| H_3_BO_3_ | 0,00043% |
| Nicotinamide | 0,00007% |
| FeSO_4_ x 7 H_2_O | 0,00095% |
| Na_2_PO_4_ | 0,134% |
| (NH_4_)_2_SO_4_ | 0,354% |
| Ethanol abs.* | 0,473% |
| Glucose 50%* | 2,381% |

*added after Autoclaving
