# Supplementary figures and images for: Biogenic polymer-based patches for congenital cardiac surgery: a feasibility study
Source: Front Cardiovasc Med. 2023 Jun 22;10:1164285. doi: 10.3389/fcvm.2023.1164285 (PMC10325621; doi:10.3389/fcvm.2023.1164285)

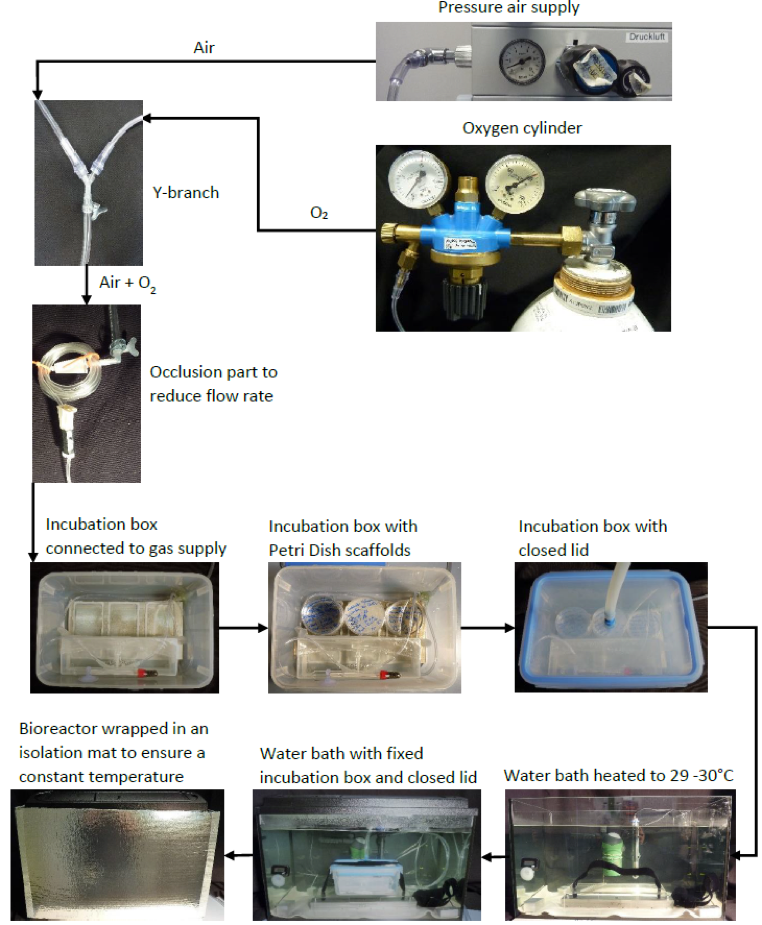

Supplement: Supplementary file 2 [file Image1.tiff]

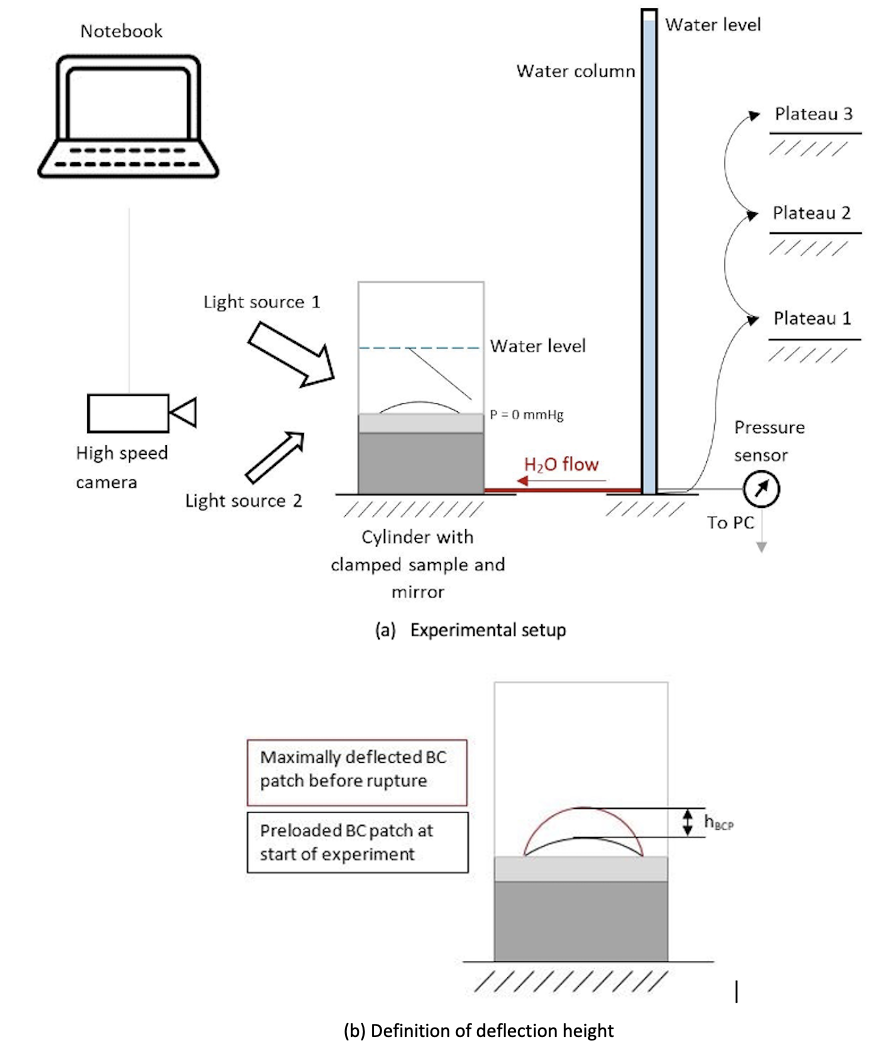

Supplement: Supplementary file 3 [file Image2.tiff]

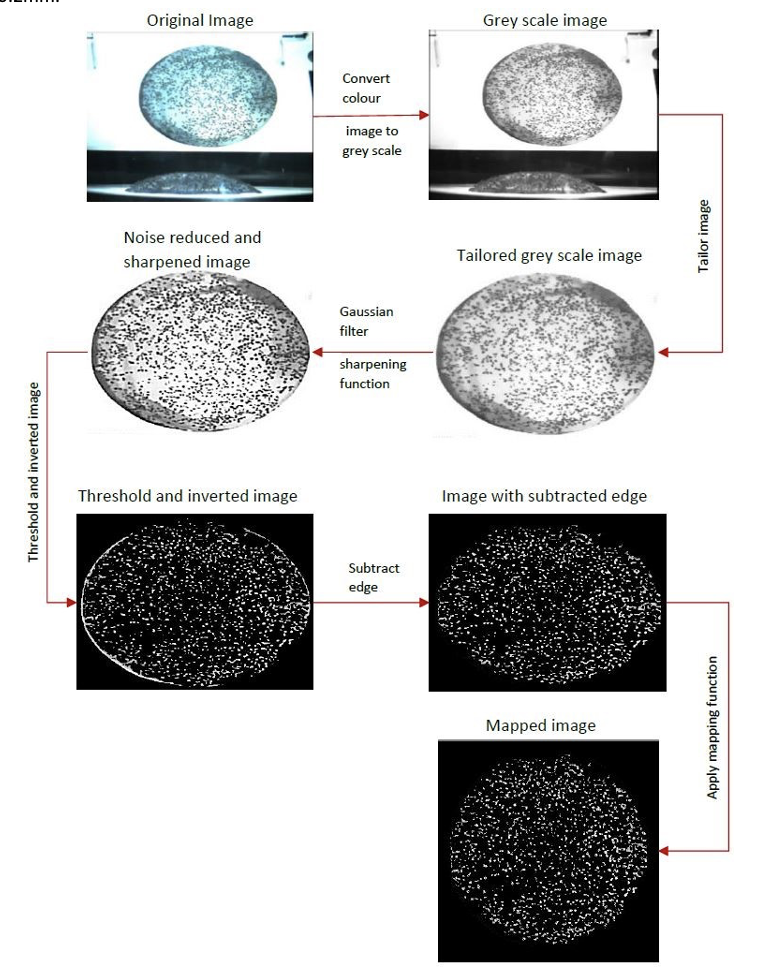

Supplement: Supplementary file 4 [file Image3.tiff]

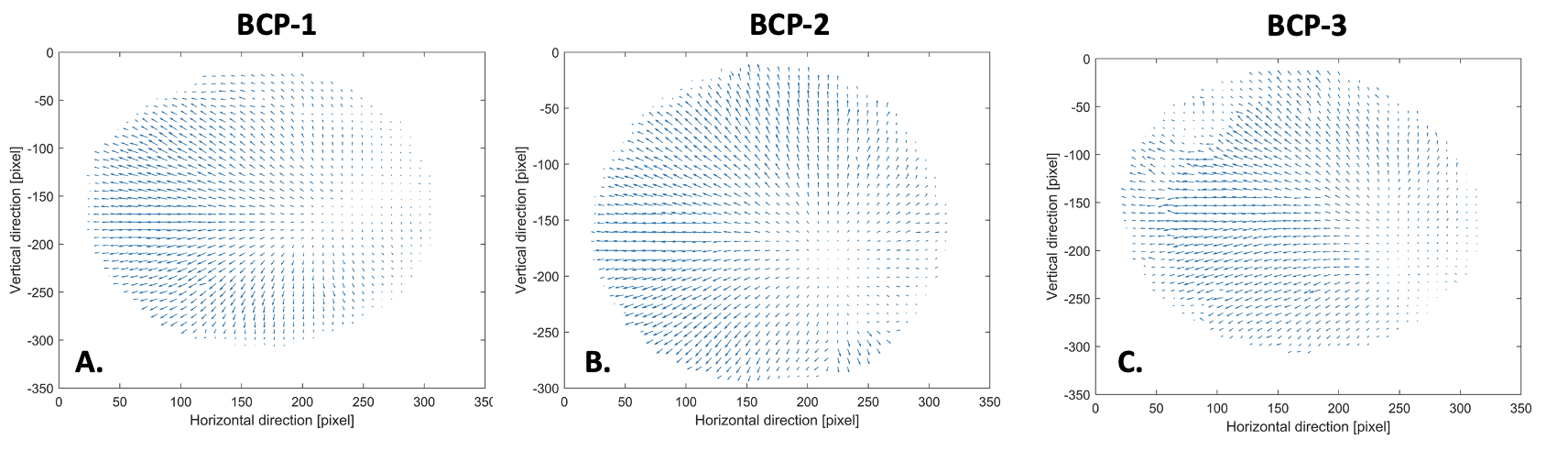

Supplement: Supplementary file 5 [file Image4.tiff]

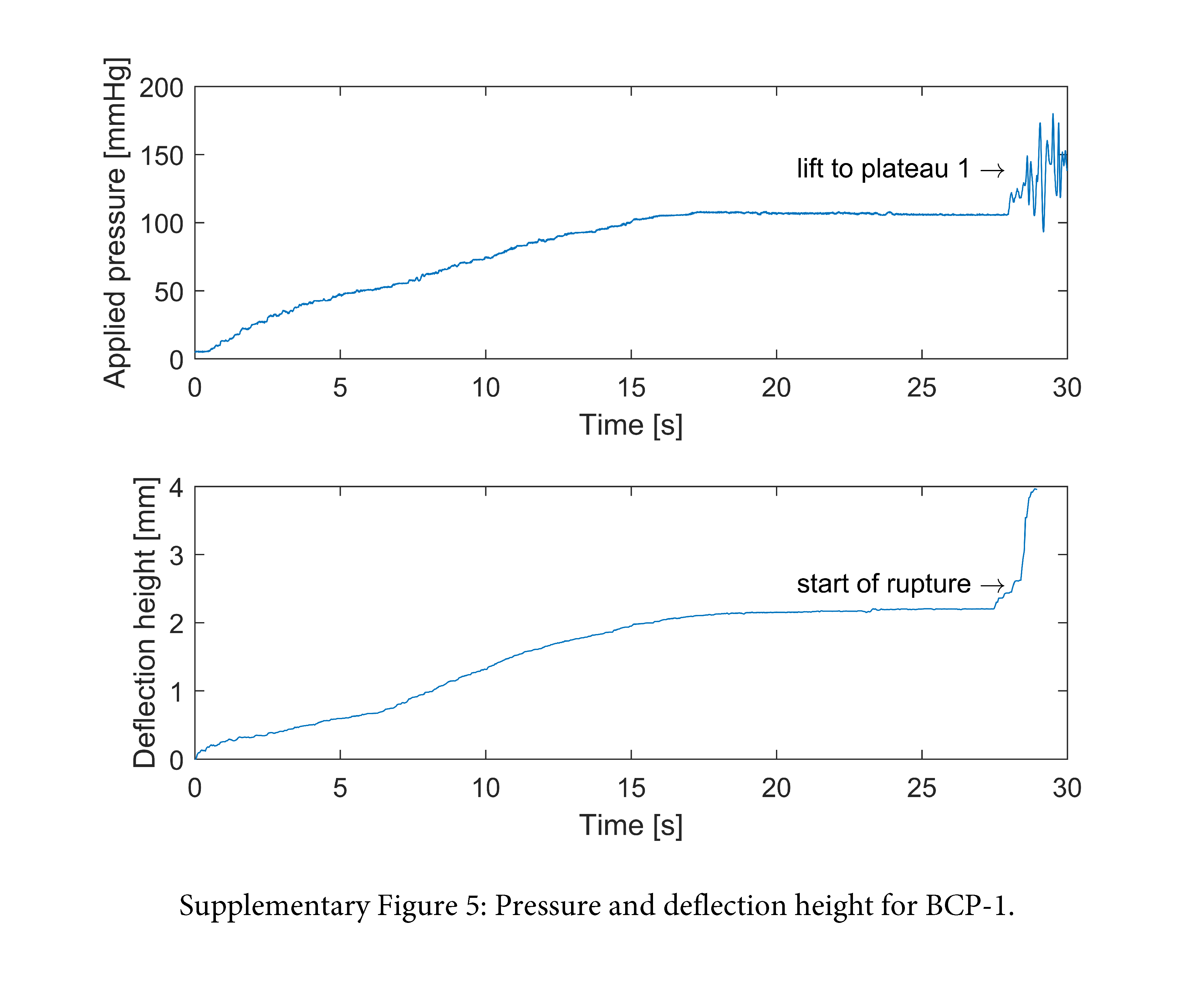

Supplement: Supplementary file 6 [file Image5.tif]

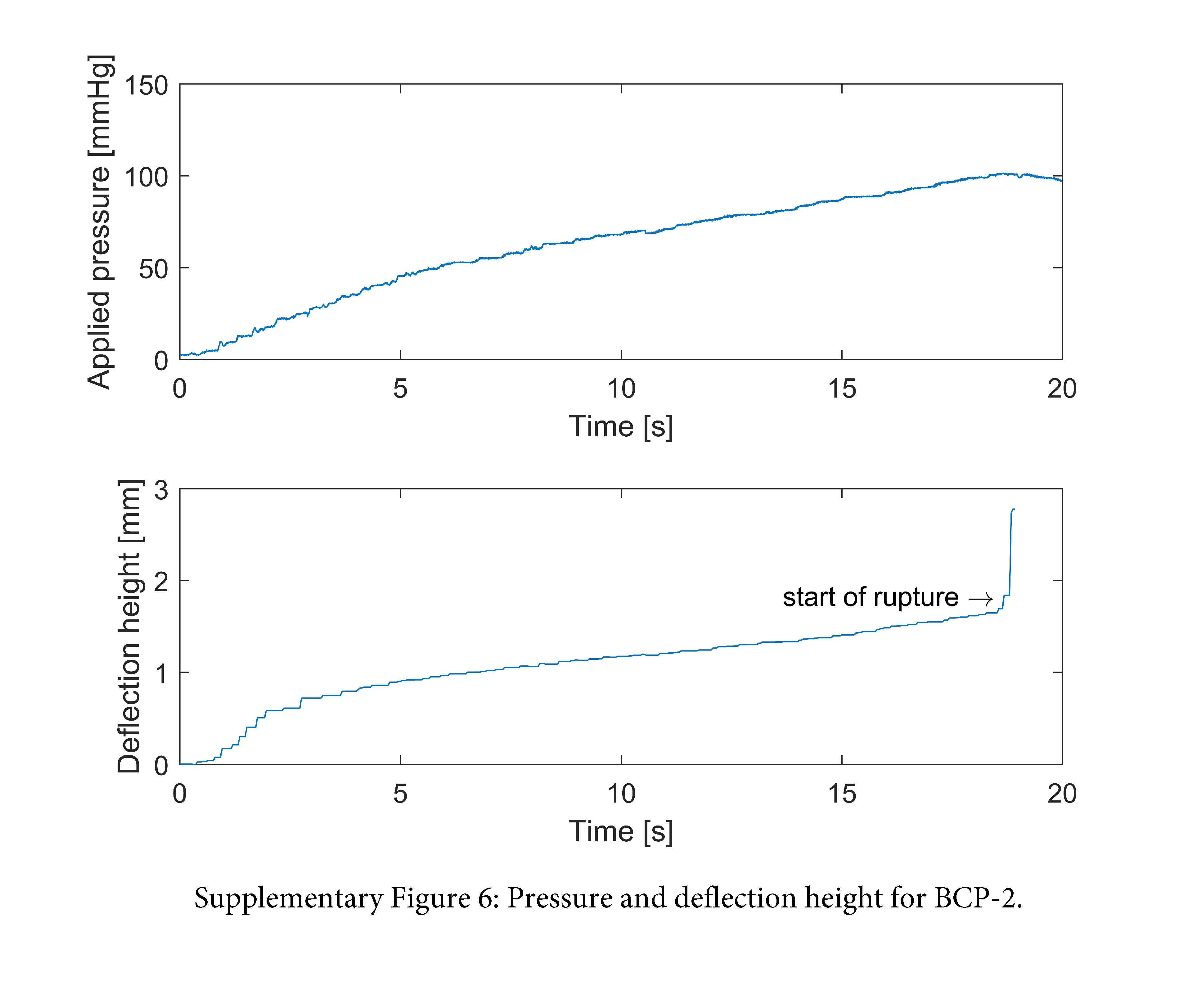

Supplement: Supplementary file 7 [file Image6.tif]

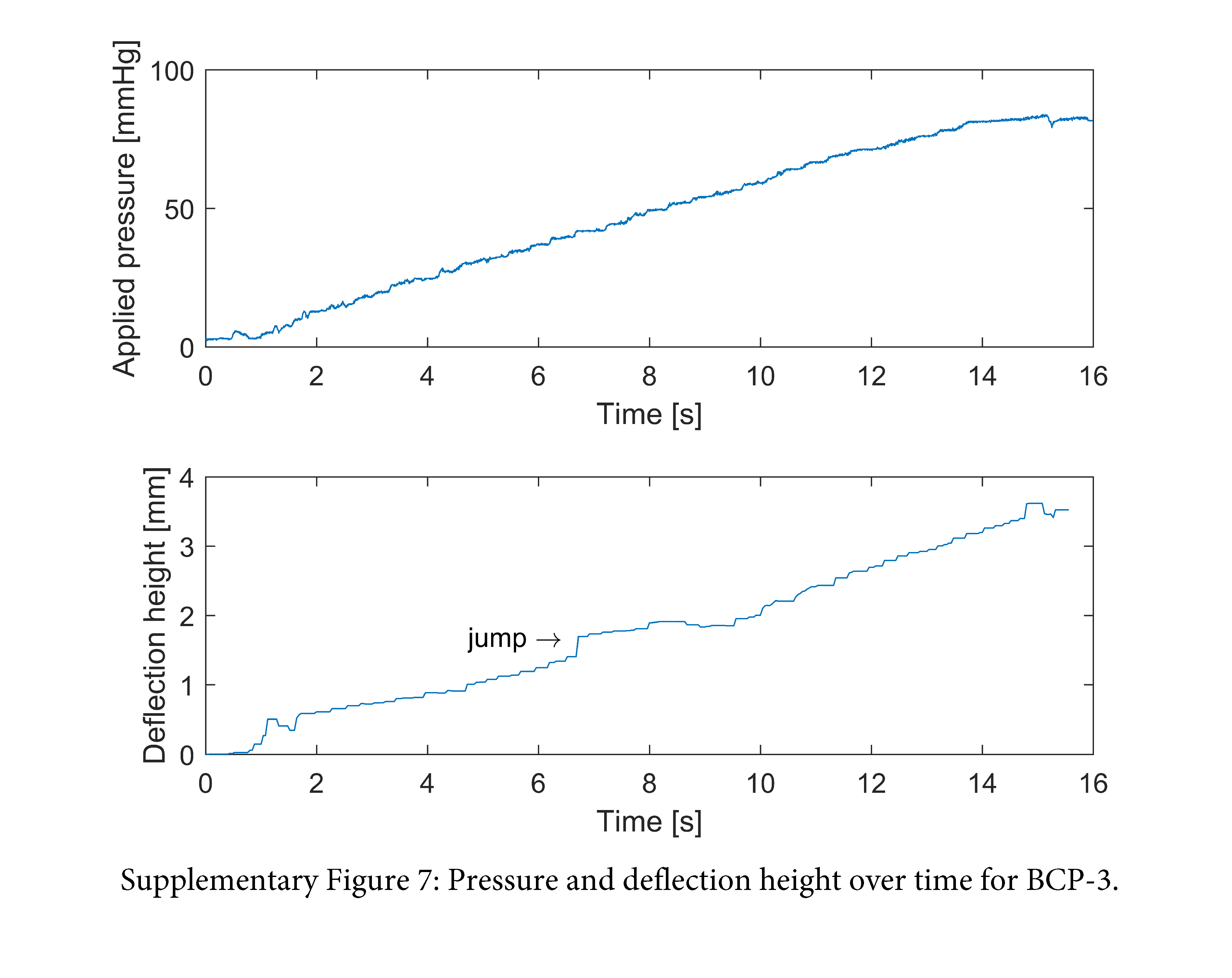

Supplement: Supplementary file 8 [file Image7.tif]

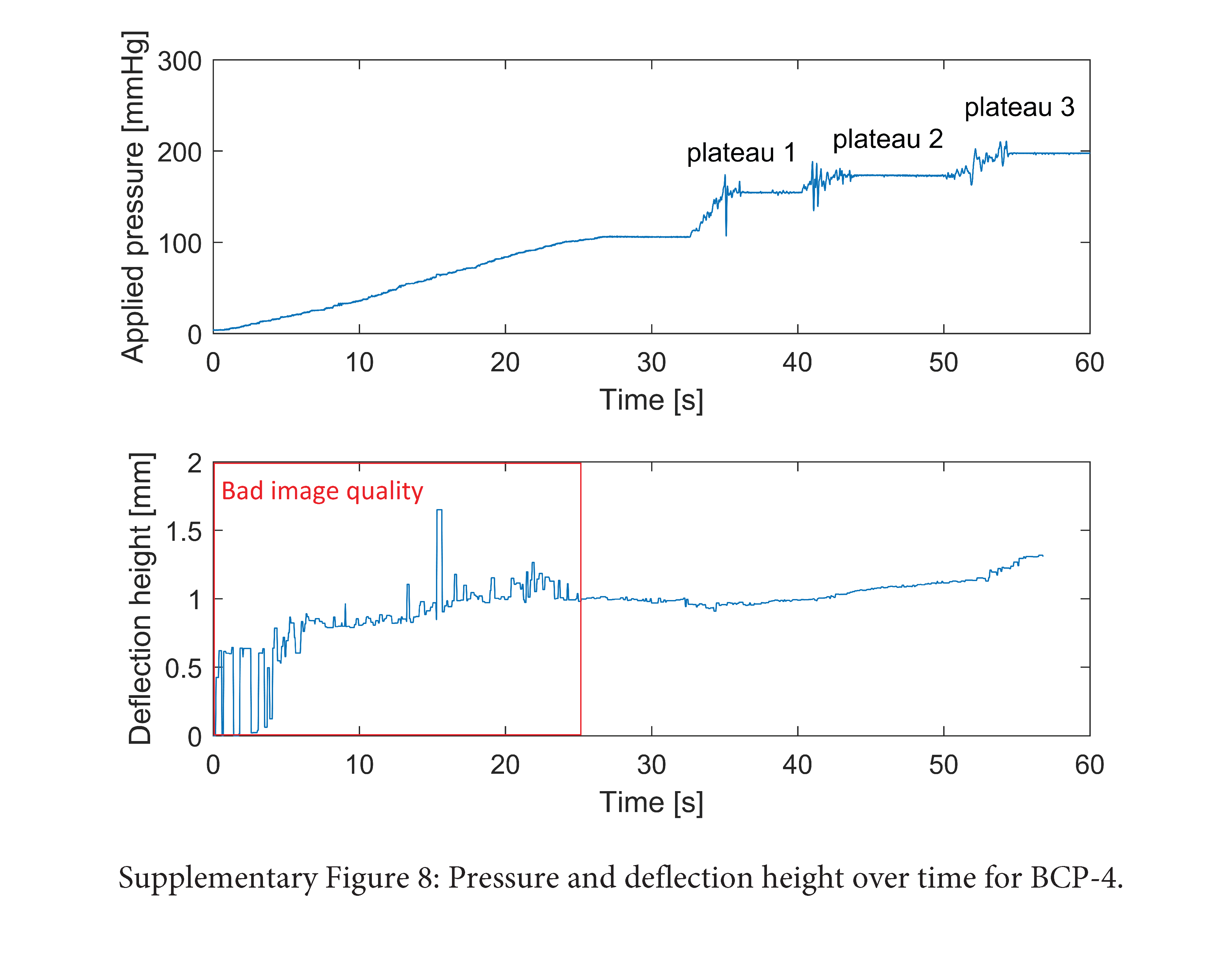

Supplement: Supplementary file 9 [file Image8.tif]

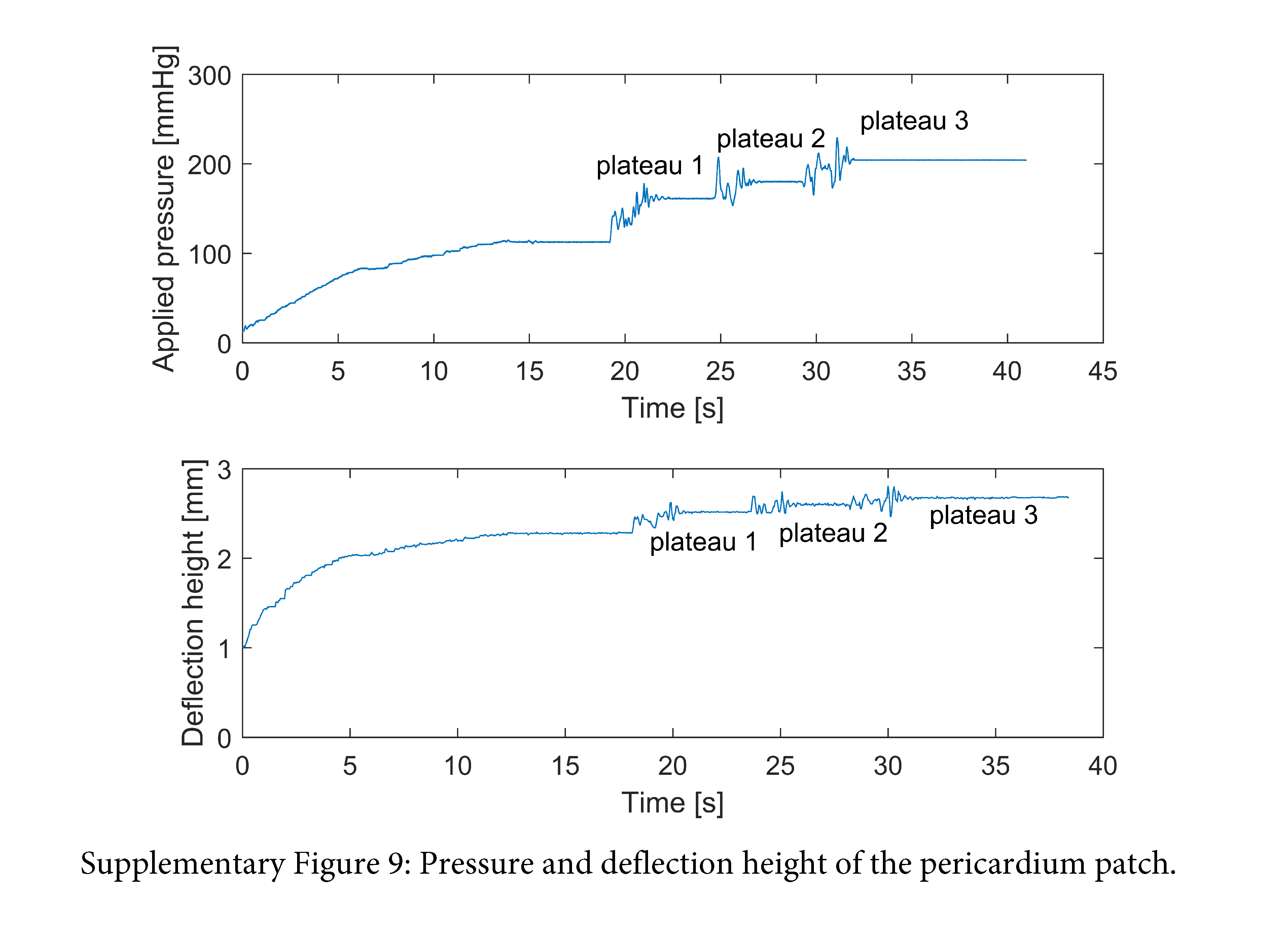

Supplement: Supplementary file 10 [file Image9.tif]
